# How to ensure provision of quality cost effective healthcare through indigenous research and rational use of modern technology

**DOI:** 10.12669/pjms.296.4345

**Published:** 2013

**Authors:** Shaukat Ali Jawaid

In his plenary talk at the EMMJ5 Medial Journals Conference organized by Eastern Mediterranean Association of Medial Editors (EMAME) in collaboration with Pakistan Associations of Medial Editors (PAME) at Karachi in 2010, Jane Nicholson from WHO EMRO laid emphasis on local, indigenous research. She further opined that journals should publish research which is relevant to the country, community, to patients and policy makers.^1^ Publishing research work which has no relevance to the country and region which the journal caters to is of no use. Different countries have their own problems and the best way to combat the diseases prevalent in their countries is through indigenous research and solutions. 

Somewhat similar views were expressed by Farrokh Habibzadeh President of World Association of Medical Editors (WAME) while speaking at the Asia-Pacific Association of Medical Editors (APAME) congress held at Kuala Lumpur Malaysia. He stated that “we should not accept all rules set by international organizations but customize them to suit our own requirements. Biomedical journals should publish research on locally prevalent diseases instead of covering more advanced subjects like molecular biology”.^2^

These both distinguished medical editors have rightly stressed the need for local research relevant to our needs and requirements. Not only that we have to think of ways and means to ensure provision of quality cost effective healthcare with rational use of modern technology. What is good for the West may not be suitable for the less developed countries with huge disease burden and financial constraints. Despite advances in modern technology, even the developed countries in the West are worried about the increasing cost of healthcare.

While these days there is lot of emphasis on evidence based medicine but it is also important that this EBM should be based on results from studies done in the region and their own countries. For example at cardiology conferences in Pakistan there is often debate as to which one is best and most suitable treatment modality for Pakistani patients suffering from coronary artery disease i.e. interventional cardiac procedures or coronary artery bypass graft surgery (CABG). The speakers emphasize on evidence based medicine and refer to various studies done in the West which show that most often interventional cardiac procedures are much more cost effective but they forget the fact that it may be true for that country but not for the developing third world countries .What we need is local evidence based medicine studies because surgery is very costly in the West as compared to the developing world where most often CABG is the cost effective strategy while managing such patients. Moreover, treatment approach in each patient has to be individualized keeping in view various factors i.e. cost of stents, balloons, interventional procedures and CABG surgery, age and health condition of the patient, co-morbid diseases and above all financial position of the patient and the family more commonly known as “pocket biopsy”. 

However, it will be interesting to note that a comparative study done in Isfahan in Iran about late clinical events of drug eluting versus bare metal stenting conducted in four hundred forty two patients, one hundred sixty six in the DES group and one hundred ninety seven in BMS group showed that prevalence of the in-hospital major cardiac events, angiographic and clinical success rate were the same between both the groups. There were no significant differences regarding six and twelve month major cardiac events in patients treated by BMS or DES. The authors concluded that considering the same clinical outcome and economical parameters, use of the BMS after proper patient selection was recommended.^3^ This is a regional evidence based medicine which is much more relevant than studies done in the West.

Another study by Aiju Xiao et al from China compared the clinical effects of surgical and Gamma knife treatments of hippocampal sclerosis-induced intractable epilepsy of children below age ten years showed that patients in both the groups were treated effectively but the surgical treatment lead to better results at a reduced cost.^4^ All this shows that we have to ensure rational use of modern technology and availability of Gamma Knife facilities in the country does not mean that one has to prefer it for every patient. Of course it could be the patient’s choice and the treating team should provide all the information about the treatment modalities available, their benefits and disadvantages along with the cost and involve the patient or his/her family members in final decision making as regards the treatment modality. 

Similarly while endoscopic thyroidectomy is the latest state of art treatment modality but it is a very difficult procedure, there are limitations of space for work, it is more invasive hence more chances of complications. On the other hand as demonstrated by Prof. Sikandar Ali Shaikh at a recent endocrine surgical course held at JPMC, open procedure is much more practical and cost effective. He further stated that the technique which he demonstrated in detail was “indicated for the unilateral benign goiter in the middle age or elderly where scar is not a problem. It is also indicated for ablation of remnant of thyroid tissue in malignant goiter where lobectomy has already been performed.” ^5^ Hence such a technique was more suitable for patients in developing countries faced with financial constraints and lack of advanced technology.

It is unfortunate that the new generation of healthcare professionals under the influence of aggressive marketing by manufacturers of sophisticated equipment and instruments have become slave of modern gadgetry. They seem to have forgotten the golden principle that detailed history and comprehensive clinical examination most often leads to correct diagnosis which can then be confirmed using diagnostic and imaging facilities. Training in clinical skills are extremely important but these days in most of the medical institutions they are neither taught to the medical students nor they are aware of its importance. 

In some cases those specialists who are trained abroad apply their knowledge to the local patients without considering the local environments and psyche. It is just like transplanting an alien organ into an alien body. Wisdom demands that second thought must be given while managing the local patients. This is also in line with selecting the research topics not necessarily relevant to our population.

In order to reduce the cost of care and eliminate the un-necessary diagnostic procedures medical organizations in the West have been participating in a campaign to help clinicians and patients avoid wasteful and at times harmful medical interventions. A group of experts recently analyzed numerous studies to improve healthcare value. Their recommendations were published by Journal of Hospital Medicine which these experts opined will reduce the cost besides leading to better care of the patients. These experts were of the view that almost 20% of the healthcare cost can be reduced by eliminating waste. “Their five recommendations for hospitalized children are as under:^6^

1. Don’t order chest radiographs in children with asthma or bronchiolitis. This has the potential to decrease costs, reduce radiation exposure, and minimize the overuse of antibiotics due to false positive results. 

2. Don’t use bronchodilators in children with bronchiolitis because the agents have minimal or no treatment effects. 

3. Don’t use systemic corticosteroids in children under two years of age with a lower respiratory tract infection because the treatment is potentially harmful and provides little or no benefit. 

4. Don’t treat gastro esophageal reflux in infants routinely with acid suppression therapy, such as proton pump inhibitors. Studies show that such treatment is no more effective than placebo in infants, and it may cause side effects. 

5. Don’t use continuous pulse oximetry—a method for measuring oxygen saturation in the blood—routinely in children with acute respiratory illness unless they are on supplemental oxygen. Continuous monitoring of oxygen saturations in hospitalized infants with bronchiolitis may lead to over diagnosis of hypoxemia, increased hospital duration, and the use of oxygen that is of no apparent benefit to the child”. 

The lead researcher Ricardo Quinonez, from the Children’s Hospital of San Antonio and Baylor College of Medicine feels that that if child specialists adopt and practice these recommendations, it could result in huge savings. 

Similarly the Society of Hospital Medicine also outlined five recommendations regarding care of the adult.^7^ These recommendations are: 

1. “Do not place, or leave in place, urinary catheters for incontinence or convenience or monitoring of output for non-critically ill patients. 

2. Do not prescribe medications for stress ulcer prophylaxis to medical inpatients unless at high risk for gastrointestinal complications. 

3. Avoid transfusions of red blood cells for arbitrary hemoglobin or hematocrit thresholds and in the absence of symptoms or active coronary disease, heart failure, or stroke. 

4. Do not order continuous telemetry monitoring outside of the intensive care unit without using a protocol that governs continuation. 

5. Do not perform repetitive complete blood count and chemistry testing in the face of clinical and lab stability.”

Writing in the same issue of Hospital Medicine in an accompanying editorial, Andrew Auerbach, and Robert Wachter of the University of California, San Francsico, stated that “The next challenge will be translating these recommendations into actionable measures and then clinical practice.”^8^

Association and specialty organizations in every country should be working on these lines and review such suggestions and modify them if need be in view of the local circumstances and then promote and ensure their implementation in clinical practice. The less developed countries and societies need such cost saving measures much more than the rich Western countries. This is the only way we can provide cost effective quality healthcare to our population and ensure judicious use of the available resources.
